# Navigating social networks in cancer care: a phenomenological exploration of patient experiences

**DOI:** 10.3332/ecancer.2026.2069

**Published:** 2026-01-30

**Authors:** Yousef Roosta, Hero Khezri, Vahid Hoseinpour, Mohamad Jebraeily, Amirhossein Rayegani, Saeed Razavi-Dizaji

**Affiliations:** 1 Department of Internal Medicine, School of Medicine, Imam Khomeini Hospital, Urmia University of Medical Sciences, Urmia 5715789397, Iran; 2 Department of Health Information Technology, School of Allied Medical Sciences, Urmia University of Medical Sciences, Urmia 5756115198, Iran; 3 Department of Emergency Medicine, School of Medicine, Imam Khomeini Hospital, Urmia University of Medical Sciences, Urmia 5714783734, Iran; 4 Department of Radiation Oncologist, Omid Medical and Research Centre, Urmia University of Medical Sciences, Urmia 5716835377, Iran; a https://orcid.org/0000-0002-5476-9718

**Keywords:** cancer, social networks, patient experiences, phenomenology, qualitative research

## Abstract

**Background:**

Social networks (SNs) are increasingly shaping the landscape of cancer care by providing online platforms for information exchange, emotional support and patient engagement. However, the nuanced experiences of patients using these platforms, along with the associated risks and expectations, remain underexplored. This qualitative study aimed to investigate cancer patients’ perspectives on the role of SNs throughout their care journey.

**Methods:**

A phenomenological design was employed in 2024, involving 20 cancer patients who were active users of SNs. Participants were purposively selected from hospitals affiliated with Urmia University of Medical Sciences, Iran. Data were collected through semi-structured interviews focusing on three main areas: perceived opportunities, challenges and expectations. Thematic analysis was conducted following Braun and Clarke’s six-phase framework.

**Results:**

Findings are presented within three main categories derived from the interview guide: opportunities, challenges and expectations. Thematic analysis revealed several key subthemes under each category. Opportunities included enhanced cancer awareness, improved communication with healthcare providers, emotional support and self-care promotion. Challenges encompassed misinformation, privacy breaches, emotional distress, social isolation and commercial exploitation. Expectations included greater provider engagement as well as improved access to reliable treatment information and social support.

**Conclusion:**

Findings highlight that while SNs offer meaningful benefits in cancer care, they also pose substantial risks. To maximise their utility and minimise harm, these platforms must prioritise content accuracy, user privacy and clinical collaboration. To be effective in health contexts, SNs should specifically address health-related issues.

## Background

Social networks (SNs) are a powerful tool with significant potential for searching and sharing information related to cancer, enhancing education, communication, engagement and empowering patients. Currently, the use of SNs is a global phenomenon with increasing prevalence [1]. Medical information on SNs can be presented in various formats, such as videos, animations and images, allowing patients with limited literacy skills to access this information as well [2]. Notably, SNs present academic and evidence-based information, citing peer-reviewed journals in 5%–20% of cases [2, 3]. Another advantage of SNs is that cancer patients can participate in open discussions and share their experiences, personal stories, feelings and opinions anonymously with others. Anonymity promotes open and unbiased communication, minimises the fear of stigma and encourages self-disclosure, leading to broader social support and empowerment [4]. SNs offer individuals with cancer the chance to acquire knowledge about coping with the disease, while also providing emotional support that alleviates feelings of isolation, depression and anxiety [5]. These networks enhance patients' understanding of their conditions, improving their capacity to manage their health effectively and consequently assisting in the self-management of their illnesses [6]. Despite the potential benefits of SNs in the care of cancer patients, their use is also associated with specific limitations. Patients who use SNs for healthcare purposes may face issues such as loss of privacy and confidentiality of patient information, the proliferation of advertisements, addiction to SNs, decreased mental well-being, the provision of low-quality and inaccurate information and the risk of jeopardising the patient-physician relationship [1, 5, 7].

The use of social media in cancer care has become a critical issue that requires continuous evaluation in order to minimise potential risks while maximising its benefits [1]. Although several studies have explored patients’ experiences with these platforms, significant research gaps remain. For example, narrative reviews such as the study by Aristokleous *et al* [1] highlight that social media serves as a powerful tool for patient education and empowerment, yet it also poses risks, including misinformation and potential harm to the physician–patient relationship. These findings underscore the importance of a more in-depth examination of both the positive and negative aspects of these platforms from the direct perspectives of patients. Moreover, existing qualitative studies are often limited by small sample sizes; for instance, Gandamihardja *et al* [8] reflected the perspectives of only two breast cancer patients. Such limitations highlight a major research gap in the current literature, as a comprehensive understanding of cancer patients’ views on the opportunities, challenges and expectations regarding the use of social media as a health information resource is still lacking. Specifically, only a limited number of studies have quantitatively assessed how patients weigh the benefits of social support and information accessibility against concerns such as misinformation or privacy breaches. This lack of knowledge hinders the development of targeted interventions to optimise the use of social media in cancer care. In response to the need for a deeper understanding of these perspectives, the present qualitative study aims to evaluate cancer patients’ views on the opportunities, challenges and expectations associated with social media use throughout their treatment journey.

## Methods

This study adopted a qualitative phenomenological approach to explore the lived experiences of cancer patients regarding their use of SN for health-related purposes. Ethical approval was obtained from Urmia University of Medical Sciences (UUoMSs) (IR.UMSU.HIMAM.REC.1403.104). All participants provided written informed consent after being assured of anonymity, voluntary participation and the right to withdraw at any time. No identifiable data were recorded. All transcripts were stored securely and were accessible only to the research team.

### Setting, participants and sampling

The study was conducted in 2024 at hospitals affiliated with UUoMSs, Iran. From a pool of 204 cancer patients who actively used SNs, purposive sampling was employed to recruit participants until theoretical saturation was reached (final *n* = 20).

### Inclusion criteria

A histopathologically confirmed cancer diagnosis, Hospitalisation at a UUoMS-affiliated hospital, and active engagement with SNs (≥3 times per week for health-related purposes). Patients who used SNs less than three times per week for health-related purposes were excluded.

### Data collection

The study aimed to deeply explore ‘three core themes: perceived opportunities of SN use for cancer-related healthcare, challenges and concerns regarding the use of SNs in cancer care and expectations from SNs within the healthcare context’.

Data were collected through in-depth, semi-structured interviews with open-ended questions. Interviews were conducted in hospital settings by a physician (one of the researchers) and a research assistant who acted as a scribe. Necessary arrangements were made with patients who had previously expressed willingness to participate. Some interviews were conducted in outpatient clinics, while others were held in inpatient departments, based on each patient’s preference.

Before each interview, participants received a clear explanation of the study's objectives and signed an informed consent form. A printed sheet containing three guiding questions was given to participants, allowing them time to reflect on their experiences. Participants were encouraged to share detailed narratives. Follow-up questions (e.g., ‘Can you elaborate?’ or ‘How did that experience affect you?’) were used to deepen responses.

Due to confidentiality concerns and patients’ conditions, no audio recordings were made. Instead, the scribe documented the responses in real time. Only the researchers and the participants were present during the interviews, which were conducted in private rooms to ensure comfort and confidentiality.

### Data analysis

Thematic analysis was conducted following Braun and Clarke's six-phase framework: **Familiarization** – repeated reading of transcripts to identify initial patterns; **Coding** – line-by-line open coding to extract meaningful units (e.g., 'fear of misinformation'); **Theme Development** – grouping codes into subthemes and overarching themes (e.g., 'heightened anxiety concerns'); **Reviewing Theme** – refining themes through team discussions and member checking; **Defining Themes** – articulating the essence of each theme using illustrative quotes; and **Reporting** – structuring findings around key themes and subthemes [9].

All interview transcripts were reviewed and found to be clear and legible, so they were not returned to participants for validation. One researcher read the transcripts multiple times to gain familiarity with the data. Four researchers collaborated to identify main concepts, generate codes and determine overarching themes and subthemes. All team members reviewed and approved the final coding framework. Data analysis was conducted using MAXQDA software, version 10.0.

## Results

### Demographic characteristics of participants

In the present study, 20 participants took part in the semi-structured interviews. The average interview length was 25 minutes. Table 1 presents the demographic and clinical characteristics of the participants. As shown in Table 1, the majority of the patients (*n* = 14, 70%) were female, and the most common age group was 40–50 years (*n* = 6, 30%). In terms of marital status, 15 participants (75%) were married. Regarding education level, the largest group (*n* = 10, 50%) had a high school diploma. The participants primarily resided in urban areas (*n* = 19, 95%). The cancer types varied among the participants, with the majority having breast cancer (*n* = 8, 40%). Other cancer types included colon (*n* = 2), Hodgkin (*n* = 2), non-Hodgkin (*n* = 2), skin (*n* = 3), AML (*n* = 2) and ovary (*n* = 1). The key findings are summarised in Table 1.

### Patients’ perceptions of SN use in cancer care

**Figure 1.**
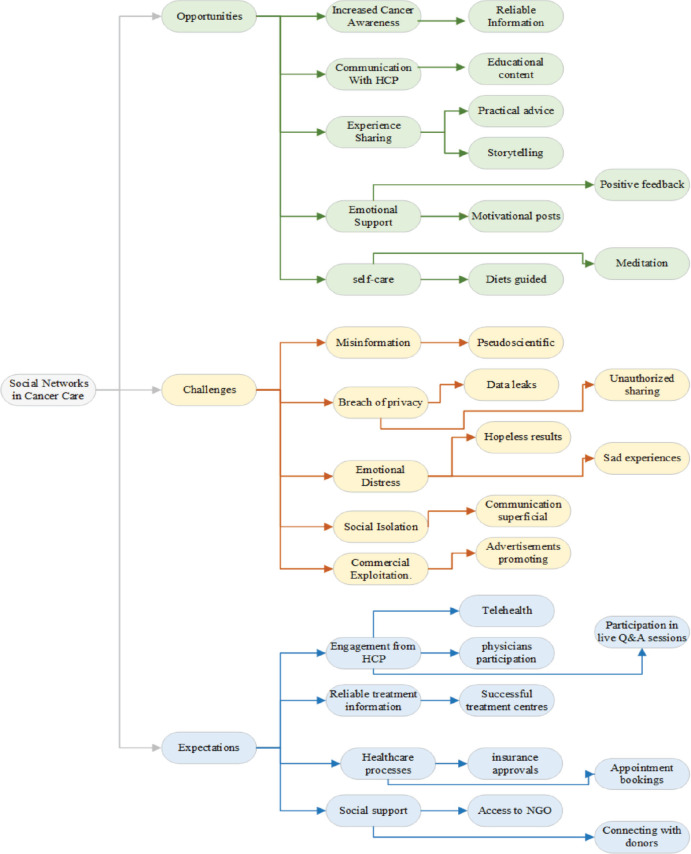
A tripartite analysis of social media from cancer patients' perspective: opportunities, challenges and expectation. Conceptual model of social media use among cancer patients, illustrating perceived opportunities (enhanced cancer awareness, communication with healthcare providers, experience sharing, emotional support and self-care promotion), challenges (misinformation, privacy breaches, emotional distress, social isolation and commercial exploitation) and expectations (greater provider engagement, access to reliable treatment information, facilitation of healthcare processes and improved social support). The model was derived from thematic analysis of 20 semi-structured patient interviews.

The findings of this study are presented within three overarching themes derived from the interview guide, reflecting the primary areas explored during the interviews. Within these overarching themes, several emerging subthemes were identified inductively from participants’ responses, providing a nuanced understanding of patients’ perspectives on SN use in cancer care. This combined approach illustrates the integration of deductive (framework-driven) and inductive (data-driven) analysis in the study. The key findings are summarised in Figure 1.

### Theme 1: opportunities of SNs in cancer care

From the patients’ perspective, the primary opportunities associated with SNs in cancer care were categorised into five key sub-themes. While these opportunities presented significant benefits, they were sometimes intertwined with complexities that patients had to navigate. The sub-themes were: increased cancer awareness, peer support and experience sharing, enhanced communication with healthcare providers, psychological and emotional support and the promotion of self-care strategies. SNs offer significant potential to increase patient awareness about cancer. Approximately four participants in this study reported accessing educational content shared by physicians, and 10 participants in this study reported accessing educational content shared by other resources (unknown), including, videos and animations that provided comprehensive information on cancer, simplified complex treatment options and offered practical guidance for managing chemotherapy side effects such as nausea and fatigue. One participant noted, ‘Instagram posts by doctors improved my understanding of chemotherapy management.’ (P03). Another patient also highlighted the value of online health information, stating, ‘I'm always looking for updated information about my illness on social media.’ (P08).

In addition to educational information, ‘participants (*n* = 10) emphasised the value of peer support and shared experiences’. Online communities facilitated meaningful storytelling, allowing patients to exchange personal journeys, practical advice and emotional encouragement. For example, some participants described coping with hair loss through shared recommendations on wigs or receiving suggestions regarding the use of prostheses following a mastectomy. One patient expressed how this support reduces feelings of isolation, stating, ‘I have followed some cancer patients, and I sometimes ask them about their experiences via direct messages. This communication makes me feel less lonely.’ (P16). Improved communication with healthcare providers emerged as another notable advantage. Several participants (*n* = 4) reported using telehealth platforms to exchange images and voice messages with their physicians. One patient recounted, ‘Through WhatsApp, I sent voice messages to my doctor and pictures of my test results when I couldn’t visit the clinic. He then issued an e-prescription and sent me a tracking code.’ (P07)

Participants (*n* = 11) also highlighted the psychological and emotional benefits of engaging with SNs. These included reduced feelings of isolation, uplift from humorous content and emotional reinforcement through motivational posts, likes and supportive comments. As one patient shared, ‘Occasionally, I scroll through my phone to look at memes and laugh—especially when I’m feeling hopeless.’ (P11)

Finally, some participants (*n* = 5) reported that SNs played a role in shaping their self-care behaviours. These included adopting exercise routines, practicing meditation and following anti-inflammatory diets guided by online dieticians. One participant explained, ‘I always choose my diet based on recommendations from a YouTube channel I subscribe to about nutrition for cancer patients.’ (P16)

### Theme 2: challenges in SN use in cancer care

Despite the potential benefits of SNs, participants identified several concerns regarding their use in cancer care. While some patients found reliable information and emotional support, others were burdened by conflicting narratives and serious risks. Thematic analysis revealed several key sub-themes: misinformation and low-quality content, emotional and psychological distress, privacy and ethical risks, social isolation and commercial exploitation.

A dominant concern among patients was the widespread dissemination of inaccurate or misleading information (*n* = 11). Participants particularly feared the promotion of unverified treatments and pseudoscientific claims, which could pose serious risks to patient safety. While most participants were aware of this danger, some also admitted that feelings of hopelessness could drive them to seek out such content. One participant shared, ‘When they discharged me from the hospital and told me to go home, I felt they had given up on my treatment. I couldn't surrender, so I started looking for miracle cures on social media. I was really just looking for a reason to have hope.’ (P01) This finding adds a crucial nuance, suggesting that the search for misinformation is often not a sign of ignorance but a desperate attempt to find hope when conventional options seem exhausted.’

‘Although there is valid information available, many videos and posts contain inaccurate or misleading content that patients may struggle to recognise as unreliable’ (P02). This burden often required participants to seek clarification from their physicians, adding another layer of effort to their care management.

Several participants also described emotional distress (*n* = 12) arising from pessimistic narratives and hopeless outcomes in other patients’ journeys and sad experiences shared online. The stories of other patients’ difficult journeys often triggered feelings of fear and despair. For example, one patient recounted.

‘I followed an Iranian cancer patient who lived in Canada. He posted hopefully about his chemotherapy, saying, ‘I will conquer cancer.’ Unfortunately, his last video showed him very ill. A few days later, I heard he had passed away, and I felt completely hopeless (P09). This sentiment was not universal, as another patient provided a contrasting view from a family perspective. They stated, ‘When I was following the pages of other cancer patients on social media, my daughter told me they are depressing and don't reflect reality, and that I shouldn't pay attention to them.’ (P20). This comment reveals the tension between the patient's need for shared experience and the family's desire to protect them from emotional distress.

Patients (*n* = 8) also raised concerns about the unauthorised sharing of medical information and the vulnerability of their data on social platforms. There were fears about potential data breaches and ethical violations related to personal content being posted without consent.

‘One patient said they felt uncomfortable when encountering videos where medical details of others had been shared. I was concerned if my physicians wanted to share my information, what could I do?’ (P14). Another participant expressed a related but distinct concern, stating, ‘When I like a post related to cancer or comment on it, I'm afraid others will find out about my illness. This worry is always with me—that my friends or acquaintances might find out about my health status through my activities on social media.’ (P11). This shows that privacy concerns are not limited to medical data but also include personal identity and social stigma.

Participants (*n* = 7) reported experiencing mental health burdens, including anxiety triggered by negative survivor stories and disrupted sleep patterns due to excessive screen time.

‘I started watching cancer-related videos at bedtime, and after several days, I noticed that I was having trouble sleepin’ (P10).

Commercial exploitation was one of the challenges in the use of SNs. Participants (*n* = 5) reported being targeted by advertisements promoting unapproved or ineffective treatments. These included ‘miracle’ cures and frequent promotional messages.

‘I was influenced by an advertisement for a herbal medicine and asked about it. After that, I received frequent promotional messages. Eventually, I consulted my doctor and decided not to purchase it’ (P05).

Patients (*n* = 6) found that relying solely on the use of SNs may lead to social isolation. While digital platforms facilitated communication, some patients felt that online interactions were often superficial and lacked emotional depth. This sometimes led to feelings of disconnection or isolation.

‘Usually, our conversations with other patients online are short. After asking a few questions and getting brief answers, I try to end the conversation because I’m not sure how they are really doing. Do they tend to continue the conversation?’ (P18). This view was in stark contrast to other participants who found meaningful peer support online.

### Theme 3: cancer patients’ expectations of SNs in cancer care

Based on the qualitative data, four main sub-themes were identified within the overarching theme of cancer patients’ expectations of SNs in Iran. These sub-themes reflect a wide and complex range of needs that often exist simultaneously within one individual, thus reflecting the coexistence of various expectations—from educational and emotional support to practical and logistical desires. These four sub-themes are:

Reliable treatment information: Eleven patients expect reliable and up-to-date treatment information, including access to lists of successful treatment centres, updates on new therapies and educational content on prevention, nutrition and managing side effects. One patient stated: ‘At the beginning of my treatment, my family and I would visit clinics or talk to acquaintances to find a doctor with a successful track record and a center with comprehensive services. Sometimes, we received conflicting information—one patient would praise a physician while another would criticise the same doctor.’ (P04). A different participant further emphasised the need for reliable information, stating: ‘I wish SNs could provide complete information, including the number of successful treatments and direct testimonials from other patients, to make choosing a doctor easier and more informed.’ (P09) Active engagement from healthcare providers: Eight patients emphasise the need for active engagement from healthcare providers on SNs, such as participation in live Q&A sessions, webinars and collaboration with trusted medical influencers. One patient underscored this desire, stating, ‘I hope doctors and treatment centers will have a greater presence in this space and provide accurate, reliable information. Educational webinars could also be very helpful.’ (P10). Another participant added, ‘I hope users will show more respect for each other and avoid spreading misinformation. We also need more precise guidance’ (P12).

Facilitation of healthcare processes: some patients’ expectations were more focused on practical, logistical support. Many (*n* = 12) sought help with facilitating healthcare processes, such as insurance approvals, medication verification and appointment bookings. A notable number of participants (*n* = 5) also expected virtual consultations, follow-ups and check-ins via video calls, viewing them as valuable for reducing travel and improving access to remote care.

However, this expectation was not without nuance. For example, some participants highlighted the convenience of these services but also expressed reservations about their effectiveness. One patient stated, ‘During the winter when illnesses are more common, I really feel like I don't need to go out for tiring insurance paperwork and waiting for hours in the doctor's office. I wish I could get an online visit and a prescription.’ (P17). This participant also added a crucial counterpoint, revealing a deeper conflict: ‘I don't trust sending my lab results and having the doctor write a prescription without a physical exam.’

I always feel like something is missing.' (P17). This sentiment highlights a key challenge: balancing the convenience of technology with the need for a trusting, in-person patient-provider relationship. Access to social support networks: Eight participants expressed the need for greater awareness of charitable organisations and social support systems, including financial aid, access to NGO resources and platforms connecting them with donors or volunteers. ‘One patient observed: ‘The cost of cancer treatment is high, and I was unaware of supportive programs and charitable services. These centers and services should be introduced and promoted through hospital channels on SNs.’ (P03).

## Discussion

**Figure 2.**
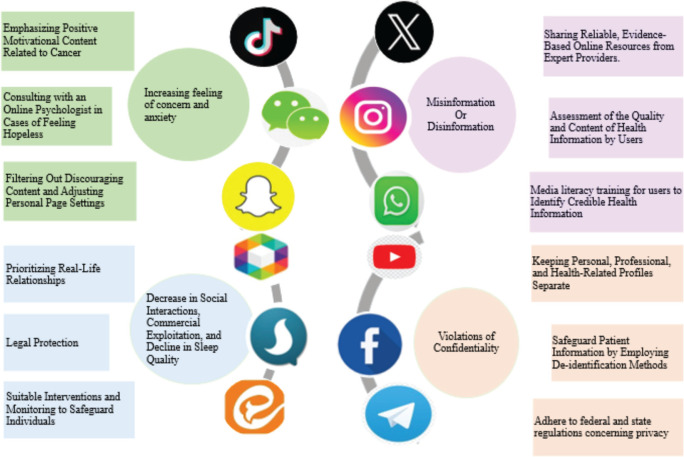
Challenges and mitigation strategies in social network-based cancer care: a conceptual framework. Conceptual mapping of key challenges in social network-mediated cancer care (central nodes) and their corresponding mitigation strategies (peripheral nodes). Strategies were derived from literature review.

The use of SNs in cancer care has expanded significantly, with patients increasingly utilising various forms of social media to interact with one another and with oncology professionals, form communities and obtain reliable information about their health or that of their loved ones [10]. While there are numerous benefits to using SNs for cancer patients, there are also valid concerns that must be addressed. Building upon these findings, our discussion delves into the specific benefits and challenges of SN use, grounded in the lived experiences of cancer patients. We also propose targeted strategies to address these concerns, highlighting our study's unique contribution to understanding the nuanced role of social media in patient empowerment and clinical communication. The key findings regarding patients’ concerns and proposed solutions are summarised in Figure 2.

### The benefits of using SNs

The results of this study indicate that participants reported that SNs have increased their knowledge and awareness about cancer. Several studies also indicate the role of SNs in empowering breast cancer patients by facilitating access to cancer-related information, enabling the exchange of medical advice and enhancing overall knowledge [1, 11]. Furthermore, participants expressed that sharing experiences with other patients through SNs is another significant benefit of using these platforms. Gandamihardja et al [8] state in their study that SNs allow individuals to connect with others facing similar challenges, such as the anxiety of waiting for scan results, fatigue and the feeling of friends distancing themselves in real life along with a multitude of other common emotions. This sharing enables fears, concerns and anxieties to be openly expressed [8].

### Concerns regarding the use of SNs and strategies to address them

#### Misinformation and unscientific information

In the current study, a number of participants expressed concern regarding misinformation and inaccurate recommendations related to cancer, including topics, such as medications and other relevant areas on SNs. The quality and reliability of medical information shared on SNs often fall short. Furthermore, this information may be unreferenced, incomplete or informal, and the authors of the medical content may remain unidentified. This issue represents one of the primary limitations of medical information disseminated on SNs [12]. The Cotter study also indicated that young women with breast cancer experienced difficulties in finding accurate and reliable online information [13]. In this context, healthcare professionals can assist by identifying and correcting misinformation and by sharing evidence-based content on SN platforms. Additionally, accurately disseminating medical research findings on SNs may be an effective strategy [14]. However, the practical challenges and workload implications of these responsibilities for healthcare professionals are discussed in the following section on patients' expectations.’

The second approach involves seeking information in a ‘step-by-step’ manner, rather than researching everything simultaneously. It is important to critically evaluate the information and describe the sources used [13]. The concept of ‘step-by-step information seeking’ is derived from conceptual models of health literacy. According to the integrated health literacy model proposed by Sørensen *et al* [15], the process of information seeking involves four key steps: accessing the information, understanding the content, critically appraising its credibility and finally applying it to make informed health decisions. This approach provides a structured framework for patients, helping them avoid the confusion that can arise from an overwhelming amount of information and enabling them to interact with online resources more effectively. Individual differences in literacy levels can significantly influence the comprehension of the information provided. Network literacy is defined as a crucial component of digital health literacy. This concept extends beyond basic computer literacy, referring to a set of skills that enables patients to function effectively in online environments, particularly on social media. These skills include critical thinking, the ability to evaluate the credibility of resources and the management of online privacy. Recent research indicates that digital health literacy, as a new determinant of health, can lead to better self-management, greater participation in medical decisions and ultimately, a higher quality of life. These skills are vital for ensuring patients have access to accurate and reliable information in the digital world [16].’ In the process of seeking health information on SNs, a shared responsibility exists between the patient and the content provider. Patients must employ their health literacy skills to critically appraise the content and evaluate the credibility of its sources. Concurrently, organisations and their official channels are responsible for proactively providing information that is transparent and credible, and for facilitating the evaluation process for patients by citing their sources. This collaborative approach helps ensure that users have access to reliable and accurate information [15, 16].

SNs and other online resources can help address these challenges through platforms that offer video-based information. However, it is essential to apply health literacy principles when designing support systems to enhance accessibility for a broad audience [17]. The assertion that video formats are more effective for improving comprehension in individuals with varying literacy levels is grounded in both Dual Coding Theory and strong empirical evidence. According to this theory and existing literature, when information is presented simultaneously through both visual and auditory channels, the barriers associated with reading skills are reduced. Consequently, SNs, by providing video-based content, offer an effective tool for promoting health literacy and access to information for a wider population, including those with limited literacy [18]. Furthermore, improving network literacy and equipping patients and their families with the necessary skills to assess the accuracy of online information is crucial for obtaining credible information. This includes relying on official websites of health organisations, hospitals and reputable research centers—such as the World Health Organisation and cancer associations—which provide reliable information [19].

#### Emotional distress

In our study, a portion of participants expressed concern about the sense of despair caused by treatment-related content, due to negative news shared on SNs. Negative comments and the unsuccessful experiences of other patients in overcoming illness adversely affect the morale of cancer patients. In a related study, researchers noted an increase in feelings of anxiety, diminished morale and emotional distress due to negative feedback as common effects of using SNs for patients engaging with health-related communities [6]. Patients on SNs have varying preferences regarding the type and amount of information they seek. When creating online support resources, it is essential to recognise these differences. Information should be clearly categorised and organised, allowing patients to choose whether to engage with potentially distressing topics, such as mortality risks associated with cancer [20]. Social media platforms, as powerful tools with a widespread impact, have a moral responsibility to proactively address challenges such as misinformation, online harassment and discrimination. This responsibility extends beyond simple oversight and involves designing platforms to prioritise user well-being and safety. This requires implementing effective policies for vetting and categorising content, removing harmful material and providing tools that empower users to engage in a safe and positive manner [21].

#### Confidentiality

In this study, several participants expressed concerns about confidentiality. Concerns regarding the use of SNs often centre on the potential negative consequences that may arise from breaches of patient confidentiality. Sharing comments, photos or videos about a patient on a networking site can lead to legal action against the patient's healthcare providers and their employers [22].

Therefore, adhering to established guidelines for SN use by healthcare providers and patients includes the following: safeguarding patient information through identity verification, avoiding discussions about specific patients, being mindful of professional licensing requirements in their state, distinguishing between personal and professional profiles, complying with federal and state privacy laws, obtaining patient consent (for healthcare providers) and utilising the most secure privacy settings available, as well as separating confidential and public profiles (for patients) [7]. Social networking sites now offer privacy settings that allow individuals to customise their profile content and determine who can view it [23].

#### Other types of concerns

In the current study, some participants declared that the use of SNs leads to decreased social interactions. The use of social networking sites is closely linked to the dynamics of relationships. Additionally, the relationships patients have with healthcare professionals can be negatively affected by their use of SNs, resulting in shorter interactions [20]. Consequently, increased engagement with social networking sites contributes to the deterioration of family bonds [24]. It is essential for individuals to recognise the potential negative impacts of networks on their relationships and to take proactive steps to mitigate these effects. This may include setting boundaries regarding network usage, practicing digital detox and prioritising real-life relationships over online interactions [25].

Commercial exploitation: Some companies exploit cancer patients to promote products or treatments, a practice that is viewed negatively by patients [26]. The protection and safeguarding of personal data is a fundamental aspect of human rights. Consequently, legal protections for victims of the misuse of personal data for commercial purposes on SNs seem essential [27].

Sleep disturbance: Sleep quality is significantly affected by excessive use of SNs, which accounts for 26% of the issue. Such overuse can negatively impact both the quality of sleep and overall rest [28]. Implementing appropriate interventions and continuous monitoring is crucial for safeguarding individuals. Strategies such as limiting screen time before bedtime are essential to prevent smartphone addiction and improve the quality and duration of sleep [29].

### Patients’ expectations for physician engagement on social media

The findings of this study reveal that cancer patients hold high expectations for the active involvement of healthcare providers on social media platforms. Participants expressed a strong desire for direct and ongoing communication with physicians through these channels expecting them to share reliable, video-based educational content (e.g., treatment explanations, recovery advice) and to respond to patient queries via comments, messages or live sessions. Some even envisioned platforms where they could submit images or videos of symptoms for remote evaluation, similar to telehealth services.

A central component of these expectations is the importance of privacy and confidentiality. Patients consistently preferred medical information that was shared or endorsed by healthcare professionals, valuing its credibility and security over algorithm-generated or user-submitted content.

However, this ideal often conflicted with patients’ actual experiences. While many envisioned social media as an extension of clinical care, they also reported frustration with the limited visibility and responsiveness of healthcare professionals online. This disconnect highlights a significant challenge: how can the healthcare system meet these expectations in a way that is both effective and sustainable?

To address this gap, the discussion regarding patients’ expectations of social media must be grounded in the realities of the healthcare system. Meeting these expectations is a multifaceted responsibility, not a burden to be borne solely by physicians. In addition to their primary duties of diagnosis, treatment and patient care, physicians often have the responsibility of training medical students and residents, which significantly increases their workload and limits the time they have for digital engagement. Beyond these systemic challenges, a cultural dimension of this issue must also be addressed. Patients require education to set realistic expectations and understand that social media platforms cannot replace professional consultation. They should not expect private and immediate answers to every medical question in post comments. Therefore, to bridge this gap, we need organisational strategies that support physicians in managed participation within these digital spaces. The involvement of social media platforms themselves and patient education are also crucial. It is this collaboration among physicians, healthcare organisations, patients and digital platforms that can create a sustainable and effective digital health care system.

### Strengths of the study

This study has several strengths that contribute to the current body of knowledge. First, by using a qualitative phenomenological approach, we were able to gain deep, rich insights into the lived experiences of cancer patients. This method allowed for a nuanced understanding of their perspectives on SNs in a way that a quantitative study could not.

Second, our focus on active social media users allowed us to collect valuable data directly from individuals who are actively navigating their health journey with the help of digital platforms. This provided firsthand accounts and detailed information about how these platforms are actually used, their benefits and the challenges faced by this specific demographic.

Third, our study addresses a significant gap in the literature by focusing on the experiences of cancer patients in Iran, a context often underrepresented in global health research. The findings offer unique insights into the role of SNs within a specific cultural and digital environment, which can inform future health interventions and support systems in similar contexts.

Finally, our commitment to participant privacy and trust by not audio-recording the interviews was a key strength. This approach fostered an environment where participants felt comfortable sharing sensitive and personal information freely, leading to more honest and authentic data.

### Study limitations

This study has several limitations. First, the sample size of 20 participants is relatively small, although it is appropriate for a phenomenological approach and we achieved data saturation after 18 interviews. Second, our recruitment method created a self-selection bias, as participants were active social media users, potentially excluding those who are less engaged or concerned with privacy. Third, our reliance on social media may have been impacted by digital inequality in Iran, potentially excluding individuals with limited digital access or literacy. Finally, the interviews were not audio-recorded to ensure participant privacy and trust, which was a deliberate choice to encourage open communication on a sensitive topic. While detailed notes were taken, the lack of verbatim transcripts is acknowledged as a limitation. These points should be considered when interpreting our findings and suggest opportunities for future research with a broader range of participants.

## Conclusion

This study highlights the dual nature of social media in the context of cancer care serving both as a valuable tool for education, support and communication, and as a source of significant challenges. On the one hand, SNs empower patients by enhancing their knowledge, providing emotional support through peer connections, and offering accessible avenues for healthcare engagement. On the other hand, concerns such as misinformation, emotional distress, breaches of confidentiality, social disconnection, commercial exploitation and sleep disturbances reveal the urgent need for responsible use and professional oversight.

Crucially, the findings underscore a growing expectation among patients for active participation of healthcare providers in digital spaces. Patients seek credible, timely and personalised information from trusted professionals, highlighting the importance of physician engagement in addressing both informational and emotional needs online. However, the gap between these expectations and current practice points to the necessity of institutional frameworks that support clinician involvement without compromising ethical standards, privacy or workload.

To optimise the use of social media in cancer care, a multi-stakeholder approach is essential. Healthcare systems, professionals, patient advocacy groups and policy-makers must collaborate to develop safe, credible and patient-centered digital ecosystems. These systems should prioritise digital health literacy, promote regulated content sharing and ensure privacy protections. By addressing existing barriers and aligning with patient expectations, SNs can evolve into trusted platforms that meaningfully complement traditional cancer care and improve patient outcomes.

## Conflicts of interest

The authors declared no potential conflicts of interest with respect to the research, authorship and/or publication of this article.

## Funding

This study received financial support from Urmia University of Medical Sciences (No. 3524). The funder had no involvement in the study design, data collection, analysis, interpretation, or manuscript preparation.

## Informed consent

Different ethical aspects of the present research were approved by the Ethics Council of Urmia University of Medical Sciences [IR.UMSU.HIMAM.REC.1403.104]. The study adhered to “ethical guidelines for anonymous survey-based research.

## Author contributions

Khezri & Roosta: Research design, overall supervision, methodology, literature review, participation in meetings, manuscript writing, Thematic analysis, Figures design and data collection collaboration.

Hosseinpour & Jebraily: Meeting participation, study coordination, data collection, manuscript editing, collaborative literature review, questionnaire design contribution.

Raigani & Razavi Dizaji: Data collection, meeting participation, and manuscript editing.

## Figures and Tables

**Table 1.** Characteristics of the participants.

Variable (*N* = 20)		Frequency (%)
Gender	Female	14 (70%)
	Male	6 (30%)
Age	10–20	1 (5%)
	20–30	5 (25%)
	30–40	4(20%)
	40–50	6 (30%)
	50–60	3 (15%)
	60–70	1 (5%)
Marital status	Single	5 (25%)
	Married	15(75%)
Education level	Less than high school diploma	1 (5%)
	High school diploma	10(50%)
	Bachelor	7 (35%)
	Master	2(10%)
Job	Housewife	7 (35%)
	Self-employed	7 (35%)
	Student	3 (15%)
	Employee	2 (10%)
	Unemployed	15 (5%)
Residential status	Urban	19 (95%)
	Rural	1(5%)
Cancer types	Breast	8 (40%)
	Colon	2 (10%)
	Hodgkin	2 (10%)
	Non-Hodgkin	2 (10%)
	Skin	3 (15%)
	AML	2 (10%)
	Ovary	1(5%)

Table sociodemographic profile of cancer patients interviewed in a semi-structured qualitative study, including age, gender, marital status, occupation, residence type and education level
